# Supplementary material for: Tau-tubulin kinase 1 and amyloid-β peptide induce phosphorylation of collapsin response mediator protein-2 and enhance neurite degeneration in Alzheimer disease mouse models
Source: Acta Neuropathol Commun. 2020 Feb 4;8:12. doi: 10.1186/s40478-020-0890-4 (PMC7001309; doi:10.1186/s40478-020-0890-4)
Supplement: Supplementary file 1 — Additional file 1: Figure S1. Representative conversion of CMYK format and extraction of Y channel (right) for DAB intensity measurement. Figure S2. Split channel images of pCRMP2 (green in bottom panels of A and B) and Dapi (blue in bottom panels of A and B) as shown in Fig. 2a and c. Scale bars = 50 (A) and 200 μm (C). [file 40478_2020_890_MOESM1_ESM.pdf]

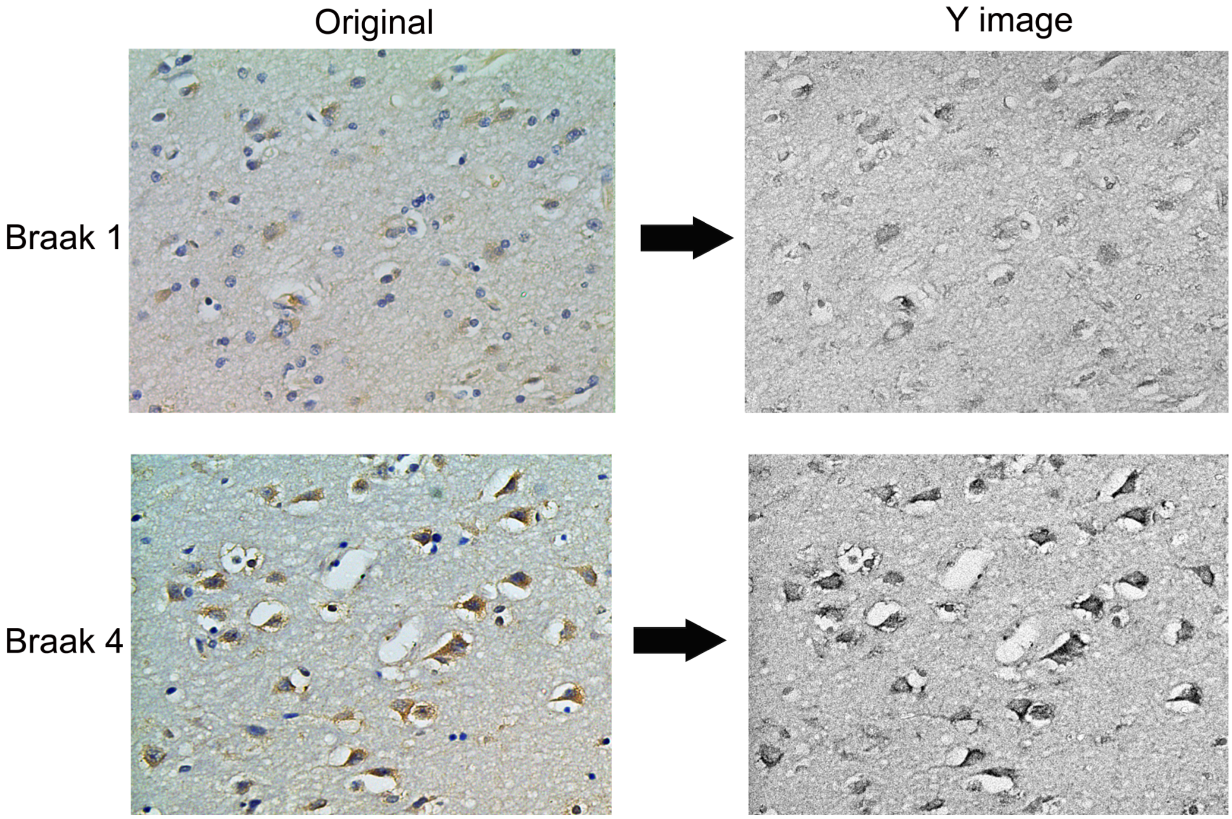

Supplementary Figure 1. Representative conversion of CMYK format and extraction of Y channel (right) for DAB intensity measurement.

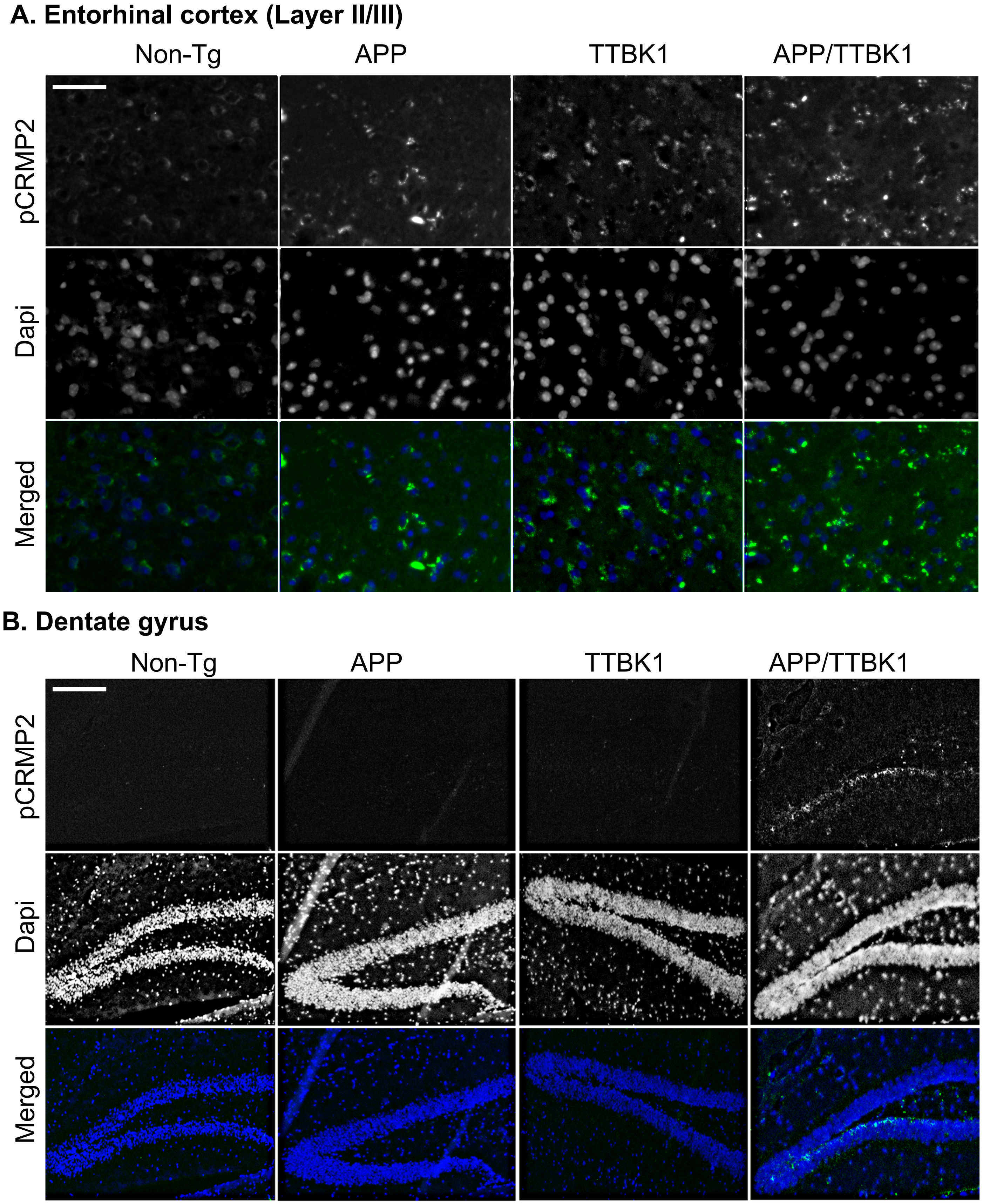

Supplementary Figure 2. Split channel images of pCRMP2 (green in bottom panels of A and B) and Dapi (blue in bottom panels of A and B) as shown in Fig. 2A and C. Scale bars = 50 (A) and 200 μm (C)
